# Phenotypic and Genomic Characterization of Bacterial Strain TAM1, a Potential Biocontrol Agent Against *Tetranychus urticae*

**DOI:** 10.3390/microorganisms14061192

**Published:** 2026-05-25

**Authors:** Shu-Chen Chang, Jianchi Chen, Chung-Chieh Lee, Ming-Yao Chiang, Hsuan Shentu, Hsien-Tzung Shih, Adalberto Á. Pérez de León

**Affiliations:** 1 Applied Zoology Division, Taiwan Agricultural Research Institute, Taichung 413008, Taiwan; cclee@tari.gov.tw (C.-C.L.); mingyaw@tari.gov.tw (M.-Y.C.); shentu@tari.gov.tw (H.S.); htshih@tari.gov.tw (H.-T.S.); 2 San Joaquin Valley Agricultural Sciences Center, Agricultural Research Service, United States Department of Agriculture, Parlier, CA 93648, USA; jianchi.chen@usda.gov (J.C.); beto.perezdeleon@usda.gov (A.Á.P.d.L.)

**Keywords:** biocontrol agent, bacterial acaricide, genome sequence, chitinase, gelatinase

## Abstract

The two-spotted spider mite, *Tetranychus urticae*, poses significant agricultural challenges due to its rapid population growth and high capacity for developing chemical resistance. This study evaluated the acaricidal activity of bacterial strain TAM1, isolated from naturally deceased mites in Taiwan. In bioassays, TAM1 caused over 90% adult mite mortality within 48 h. Infected mites showed symptoms of body darkening and deformation of the ventral abdominal crest lines. Enzymatic analysis confirmed significant chitinase and gelatinase activities. Whole genome sequence of TAM1 was acquired with a 5,066,903 bp circular chromosome (CP120954) and a 164,574 bp circular plasmid (CP120955). Refined functional profiling identified a sophisticated enzymatic arsenal including core chitin-active families (GH18, GH20, AA10) and 157 proteases, with a high prevalence of metallopeptidases that correlate with the detected gelatinase activities. Secretome analysis predicted 42 extracytoplasmic proteases primarily utilizing the Sec-dependent pathway, while the presence of multiple CBM50 modules suggests a potential for targeted substrate anchoring. These genomic insights provide a plausible molecular basis for the observed enzymatic potential and the localized ultrastructural disruption of the *T*. *urticae* cuticle. The alignment between phenotypic observations, microscopic evidence, enzymatic activities, and genomic data suggests that TAM1 utilizes synergistic, multi-target mechanisms to exert its acaricidal effects. Based on analyses of whole-genome sequence and 16S rRNA gene sequence, TAM1 was tentatively designated as a strain of *Kosakonia sacchari*. The bacterial strain reported here represents a promising microbial agent for integrated pest management (IPM) programs.

## 1. Introduction

The two-spotted spider mite, *T*. *urticae* Koch (Acari: Tetranychidae), is a globally distributed and highly polyphagous pest, capable of infesting over 1100 plant species, many of which are economically significant crops [1,2]. Since its first reported invasion in Taiwan during the late 1970s, it has been responsible for substantial crop losses [3]. In Taiwan’s subtropical climate, consistently high temperatures facilitate the rapid, year-round, proliferation of *T. urticae*. Managing these populations effectively necessitates frequent applications of chemical acaricides; however, the mite’s short life cycle often leads to the rapid development of chemical resistance [4]. This reflects a global trend where 558 cases of resistance to 96 active ingredients have been reported [5,6], underscoring the urgent demand for novel biocontrol agents.

Microbial pesticides offer a strategic advantage due to their multi-target modes of action, which significantly delay resistance development compared to single-site chemical acaricides [7]. Among these, entomopathogenic fungi represent a commercially mature group of biological control agents; as of 2023, a total of 27 fungal species have been registered as biopesticides worldwide [8]. In particular, *Beauveria bassiana* and *Metarhizium anisopliae*, are well-established biological acaricides with documented efficacy in penetrating the mite cuticle via specialized infectious hyphae [9].

The application of bacteria against *T. urticae* is a rapidly emerging field that complements existing fungal interventions [10,11]. It has been proposed that the high mortality induced by strains such as *Pseudomonas putida* biotype B and *Acinetobacter* sp. is potentially linked to cuticular adhesion and enzymatic degradation [12,13]; however, empirical evidence characterizing these specific mechanisms remains scarce. In addition to the characterized secondary metabolites of *Bacillus velezensis* W1 and *Pseudomonas fluorescens* isolate Q036B [14,15], specific taxa such as *Chromobacterium subtsugae* strain PRAA4-1 and *Burkholderia* spp. strain A396 have already been developed into commercial miticides, demonstrating significant efficacy against various spider mites [16].

Despite these global developments, the availability of commercial microbial acaricides in Taiwan remains severely limited, currently, *Photorhabdus luminescens* strain 0805-P2R is the sole microbial agent registered and commercially available for the control of spider mites [17]. The discovery of indigenous microbial strains offers a strategic advantage, as these organisms are naturally selected for persistence within the local ecosystem and are pre-adapted to unique environmental stressors. Among potential candidates, the genus *Kosakonia*—specifically the *Kosakonia sacchari* group—has emerged as a versatile group of bacteria with documented roles in plant-microbe interactions, yet their potential as entomopathogens remains largely under-explored [18,19,20]. Furthermore, compared to fungal pathogens, the specific genetic and enzymatic drivers of bacterial acaricidal activity remain less understood, necessitating detailed genomic characterization.

Here, we report the isolation and characterization of the bacterial strain TAM1 from naturally deceased *T*. *urticae* specimens collected in Taiwan. Unlike studies focused solely on mortality, this research integrates phenotypic bioassays with whole-genome sequencing to explore the potential genetic mechanisms—including identified chitinolytic and proteolytic repertoires—that may contribute to its acaricidal activity. By characterizing the genomic and ultrastructural features associated with its pathogenicity, we provide the foundational evidence required to further evaluate TAM1 as a potential indigenous alternative for the sustainable management of *T*. *urticae*.

## 2. Materials and Methods

### 2.1. Plants and Mites

Seeds of common bean (*Phaseolus vulgaris* var. communis *Aeschers*) were soaked overnight and germinated in seedling trays. Two-week-old seedlings were transplanted into 0.5 L pots and maintained in a greenhouse. A colony of two-spotted spider mites, maintained by the Taiwan Agricultural Research Institute (TARI) following multiple collections from various locations in Taiwan since the late 20th century, was used. Mites were reared on *P. vulgaris* seedlings in a controlled room at 25 ± 2 °C, 70 ± 10% RH, under a 12:12 h light–dark cycle. When plants became heavily infested, fresh seedlings were added adjacent to allow mite migration.

### 2.2. Bacterial Isolation

Ten naturally deceased *T*. *urticae* specimens, which exhibited a characteristic brownish-black body darkening, were collected in Taichung, Taiwan (24°01′44.9″ N, 120°41′40.2″ E). To preserve mite-associated microbiota, specimens were rinsed twice with sterile water and blotted dry without chemical disinfection. The mites were then homogenized in 99 μL of sterile water using a micro-pestle in a 1.5 mL tube. The homogenate was spread onto Nutrient Agar (NA) and incubated at 37 °C for 16 h.

Although 20 bacterial isolates were obtained, they exhibited nearly identical colony morphotypes. To identify the most potent candidates among these morphologically similar strains, a preliminary mortality screening was conducted on all 20 isolates using the bioassay protocol described in Section 2.3. Based on the screening results, the five most virulent isolates were selected for molecular identification. As 16S rRNA sequencing (Section 2.9) confirmed these isolates were conspecific, strain TAM1 was designated as the representative for all subsequent enzymatic, taxonomic, and genomic characterizations.

### 2.3. Acaricidal Bioassays

#### 2.3.1. Comparative Acaricidal Efficacy Assay

Bioassays followed a modified method of Van Leeuwen et al. [21]. Twenty adult female mites were transferred onto 2 cm diameter bean leaf discs placed abaxial side up on moist non-woven fabric in 3.5 cm Petri dishes. Bacterial cultures were prepared in nutrient broth (NB) and tryptic soy broth (TSB) as separate treatments to assess the influence of media composition on bioactivity. Strains were incubated at 30 °C and 200 rpm for 16 h, then diluted 1:100 in sterile water to achieve final concentrations of approximately 1.2 × 10^7^ CFU mL^−1^ for the NB treatment and 5.3 × 10^7^ CFU mL^−1^ for the TSB treatment.

Three negative controls—1:100 diluted sterile NB, sterile TSB, and distilled water—were included to exclude media or handling effects. Commercial acaricides, abamectin (2%) and etoxazole (10%), were purchased from Sinon Corp. (Taichung, Taiwan) and included as positive references. The concentrations used, 10 μg/mL for abamectin and 25 μg/mL for etoxazole, correspond to the recommended field doses for *T. urticae* control in Taiwan (e.g., for use on green onion, eggplant, and strawberry).

Leaf discs were sprayed with 600 μL of bacterial suspension using a Potter precision spray tower at 0.35 kg/cm^2^. Each treatment consisted of six replicates. Mortality was recorded at 24 and 48 h, and data were analyzed using the Kruskal–Wallis test followed by Dunn’s post hoc test (*p* < 0.05) in R (version 4.5.3, R Core Team, Vienna, Austria) [22].

#### 2.3.2. Dose–Response Analysis

To determine the lethal potency of strain TAM1, dose–response assays were conducted using a series of bacterial concentrations. The relationship between OD_600_ and CFU mL^−1^ was established via plate counting of the same cultures used in the experiments to ensure precise dosage. Mortality data at 48 h post-treatment were pooled across five replicates per concentration (total *n* = 100 per concentration). In instances where control mortality exceeded 5%, the data were corrected using Abbott’s formula.

Dose–response relationships were analyzed using Probit regression based on log_10_-transformed concentrations. Only concentrations yielding partial mortality (between 10% and 95%) were included in the Probit analysis to ensure model reliability. The median lethal concentration (LC_50_), LC_90_ values, and their respective 95% confidence intervals (CI) were estimated. The goodness-of-fit of the model was evaluated using the chi-square (*χ*^2^) test. When the chi-square test was significant (*p* < 0.05), a heterogeneity factor (*h* = *χ*^2^/*df*) was applied to adjust the 95% CI. All statistical analyses were performed using the ‘MASS’ (version 7.3-65) and ‘stats’ (version 4.5.3) packages in R (version 4.5.3, R Core Team, Vienna, Austria).

### 2.4. Microscopy Analysis

Bacterial suspensions of strain TAM1 prepared as described in Section 2.2 were adjusted to a final concentration of 1 × 10^8^ CFU (colony forming unit) mL^−1^. Mite cadavers treated with TAM1 in TSB for 24 h and untreated control mites were examined using both light microscopy and scanning electron microscopy (SEM). Light microscopic observations were conducted using a compound microscope equipped with phase-contrast and differential interference contrast (DIC) optics (Axio Scope A1, Zeiss, Oberkochen, Germany).

Two sample preparation methods were employed prior to SEM observation. In the first approach, cryogenic SEM (Cryo-SEM), mites that died following TAM1 treatment were directly mounted on an ultra-cool stage maintained at −30 °C (Deben, Woolpit, UK) and examined using a SEM (Hitachi SU3900, Tokyo, Japan); control mites were killed by freezing and processed in the same manner.

In the second approach (Fixative-SEM), mites were fixed overnight in 2.5% glutaraldehyde in 0.1 M PBS (pH 7.2), rinsed three times with PBS, dehydrated through a graded ethanol series of 30, 50, 70, 80, 90, 95, and 100% (5 min per step). Dehydrated specimens were then transferred to a 1:1 (*v*/*v*) ethanol:hexamethyldisilazane (HMDS) mixture for 10 min, followed by two changes of 100% HMDS for 10 min each, and air-dried in a fume hood for 10 min. Dried specimens were mounted on aluminum stubs, sputter-coated with gold for 50 s, and examined by SEM.

### 2.5. Morphological and Physiological Characterization of Strain TAM1

Strain TAM1 was streaked onto NA plates and incubated at 37 °C overnight to examine colony morphology. Hemolytic activity was assessed by streaking cultures onto tryptic soy agar supplemented with 5% (*v*/*v*) sheep blood, followed by incubation at 37 °C overnight.

Cell morphology was examined under the compound microscope equipped with DIC optics (see Section 2.4) after Gram staining.

For SEM observation of bacterial cells, a 2 mL aliquot of culture prepared as described in Section 2.2 was centrifuged at 3000× *g* for 10 min. The resulting pellet was resuspended in 100 μL of 0.1 M PBS. The suspension was deposited onto 15 min pretreated coverslips coated with 0.01% poly-L-lysine and allowed to adhere for 10 min. Samples were subsequently fixed, dehydrated, and gold-coated as described in Section 2.4.

For Cryo-SEM observation of TAM1 cells on the mite surface, individuals were positioned ventral side up and mounted onto carbon adhesive tape attached to the specimen holder. A thin layer of insect adhesive was applied to further secure the mites in place. A 0.6 mL aliquot of TAM1 culture, prepared as described in Section 2.2, was sprayed onto the mites using a Potter precision spray tower. The treated mites were maintained at 25 °C for 4 h prior to Cryo-SEM examination to evaluate the distribution and attachment of TAM1 cells on the mite cuticle.

### 2.6. Growth Curve Determination

TAM1 was pre-cultured in TSB at 30 °C with shaking at 200 rpm for 16 h. The culture was adjusted to an OD_600_ of 0.1, and 20 mL was centrifuged at 18,000× *g* for 10 min. The pellet was resuspended in 20 mL fresh TSB, thoroughly mixed, and transferred into 50 mL Erlenmeyer flasks for incubation at 30 °C, 200 rpm. Aliquots (1 mL) were collected at 2, 4, and 6 h, and at 1, 2, 3, 4, 7, 9, and 14 days post-inoculation. Samples were serially diluted, plated on TSA, and incubated for colony enumeration. CFU were determined and used to generate the bacterial growth curve. Data represent mean ± SD of three independent replicates.

### 2.7. Effect of Temperature on Bacterial Growth

Effect of temperature on the growth of strain TAM1 was assessed as follows. TAM1 was pre-cultured in TSB at 30 °C with shaking at 200 rpm for 16 h. The culture was adjusted to an OD_600_ of 0.085, and 20 mL aliquots were incubated in 50 mL Erlenmeyer flasks at 15, 20, 25, 30, 35, 40, and 45 °C with shaking at 200 rpm for 4 h. Following this 4 h incubation, cultures were serially diluted and plated on TSA to determine CFU. Data were transformed as necessary to meet the assumptions of normality and homogeneity of variance before analysis. Bacterial counts were square root transformed prior to analysis. One-way ANOVA followed by Fisher’s least significant difference (LSD) test was performed at a significance level of *p* < 0.05. All analyses were performed using SAS Enterprise Guide 7.1 (SAS Institute, Cary, NC, USA).

### 2.8. Enzyme Activity Assays

#### 2.8.1. Bacterial Culture Preparation

TAM1 cells were cultured on tryptic soy agar (TSA) at 30 °C for 16 h, scraped, resuspended in sterile water, and adjusted to an optical density of OD_600_ = 0.6. A 10 mL aliquot was inoculated into 70 mL of TSB and incubated at 30 °C with shaking (200 rpm) for 16 h. All treatments were performed in triplicate.

#### 2.8.2. Chitinase Assay

To differentiate between secreted and intracellular enzymes, chitinase activities were measured in both the culture supernatant and the cell lysate. Bacterial cultures (70 mL) were centrifuged at 5000× *g* for 10 min. The resulting supernatant was concentrated 100-fold using a 10 KDa molecular weight cut-off (MWCO) centrifugal filter. For the extraction of intracellular enzymes, cell pellets were frozen at −80 °C overnight, resuspended in 1.4 mL of lysis buffer, and treated with lysozyme (350 µL, 20 mg mL^−1^) at 30 °C for 30 min, followed by DNase and RNase (70 µL each) at 4 °C for 30 min. The lysates were then centrifuged at 16,000× *g* for 30 min at 4 °C, and the supernatants (representing the intracellular fraction) were collected.

The activities of β-N-acetylglucosaminidase, chitobiosidase, and endochitinase were determined according to the method of Nawani et al. [23], with modifications by Chang et al. [24], using the Chitinase Assay Kit (CS0980, Sigma-Aldrich, St. Louis, MO, USA). Each reaction consisted of 20 µL of model substrate and 20 µL of bacterial extract, incubated at 37 °C for 30 min, and terminated with 80 µL of 0.4 M sodium carbonate. The following substrates were used:

- 4-nitrophenyl N-acetyl-β-D-glucosaminide (β-N-acetylglucosaminidase activity)
- 4-nitrophenyl N,N′-diacetyl-β-D-chitobioside (chitobiosidase activity)
- 4-nitrophenyl β-D-N,N′,N″-triacetylchitotriose (endochitinase activity)

Absorbance was measured at 405 nm, using p-nitrophenol (0.01 µmol mL^−1^) as the standard. All assays were performed in triplicate. To ensure consistency with the concentrations used in the acaricidal bioassays (see Section 3.1.1), enzymatic activities were normalized to the bacterial cell density rather than total protein content. Results are expressed as U per 10^9^ CFU.

#### 2.8.3. Gelatinase Activity Assay

Gelatinase activity was assayed using gelatin as the substrate following McLaughlin and Weiss [25]. To ensure terminological consistency, ‘gelatinase activity’ is used throughout this study to refer to this proteolytic capacity against collagen-derived substrates. Leucine standards (1–150 mM) were prepared in sterile water. Bacterial supernatants were incubated with 50 mM Tris–HCl (pH 7.5), 0.36 mM CaCl_2_, and 5 mg mL^−1^ gelatin at 37 °C for 30 min, with PBS as the control. Reactions were terminated by heating at 90 °C for 10 min.

The α-amino acid content was determined following Benjakul and Morrissey [26] by adding 0.2 M phosphate buffer (pH 8.2) and 0.01% (*v*/*v*) TNBS (2,4,6-trinitrobenzenesulfonic acid), incubating at 50 °C in the dark for 15 min, and using distilled water as a blank. The reaction was stopped with 0.1 M sodium sulfite, cooled to room temperature, and absorbance was measured at 420 nm. Results were expressed as L-leucine equivalents after blank correction.

To ensure standardized reporting across all assays and maintain consistency with the bioassay parameters (Table 1), gelatinase activity was expressed solely as µmol L-leucine equivalents released per 10^9^ CFU. This normalization to cell density, rather than protein content, provides a direct correlation between bacterial concentration and enzymatic potential. All assays were performed in triplicate (*n* = 3).

### 2.9. 16S rRNA Gene Sequencing and Analysis

DNA was extracted from a single colony using the DNeasy Blood & Tissue Kit (QIAGEN, Hilden, Germany). The nearly complete 16S rRNA gene was amplified using PCR with primers 27F (5′-AGAGTTTGATCCTGGCTCAG-3′) and rD1 (5′-AAGGAGGTGATCCAGCC-3′) [27]. The amplification of the almost-complete 16S rRNA gene was conducted using the Advantage^®^ 2 PCR kit (Clontech, Mountain View, CA, USA). PCR reactions were performed in a 20 µL reaction mixture composed of 3 µL of purified DNA, 2 µL of Advantage 2 PCR buffer (10×), 0.5 µL of dNTPs, 0.5 µL of Advantage 2 polymerase mix (50×), 0.5 µL of each primer, and 13 µL of sterile water. The PCR conditions consisted of an initial denaturation of 1 min at 95 °C; 35 cycles of 30 s at 95 °C, 80 s at 54 °C, 80 s at 68 °C, and a final extension at 68 °C for 3 min. Amplicons were cloned into the PCRII-TOPO plasmids using the TOPO TA Cloning kit (Invitrogen, Waltham, CA, USA). Subsequent sequencing was conducted at Tri-I Biotech Inc. (Taipei, Taiwan) using an ABI 3730 DNA autosequencer (Applied Biosystems, Waltham, CA, USA). Sequences were queried against the National Center for Biotechnology Information (NCBI) nr database via BLASTn (version 2.17.0).

### 2.10. Whole Genome Sequencing and Analysis

Genomic DNA of strain TAM1 was extracted with the Genomic-tip 20/G Kit (Qiagen, Germany) and sequenced using Oxford Nanopore (long-read; Oxford Nanopore Technologies plc, Oxford, UK) and Illumina MiSeq (short-read; Illumina, Inc., San Diego, CA, USA) platforms at Tri-I Biotech Inc. (Taipei, Taiwan). Illumina libraries were prepared and sequenced in paired-end mode (2 × 301 bp). Low-quality reads (Q < 20) and adapters were trimmed using Qiagen CLC Genomic Workbench v10. Reads were assembled de novo with SPAdes v3.13.0 [28],. Genome annotation was performed with the NCBI Prokaryotic Genome Annotation Pipeline (PGAP) [29], and proteins were classified using the COG database. Taxonomic identity was assessed by average nucleotide identity (ANI) using TAM1 genome against the genomes of candidate strains based on the results of 16S rRNA BLASTn analysis (fastANI) [30].

Putative chitinolytic genes were identified from sequence annotations using chitin as a keyword and queried against the NCBI non-redundant (nr) database for BLASTn analysis. To further characterize these enzymes, functional assignments were reconciled with the CAZy (Carbohydrate-Active EnZymes) database by retrieving official annotations specifically curated for the TAM1 genome (available under the *K. sacchari* TAM1 entry). This ensured the classification of genes into glycoside hydrolase (GH), auxiliary activity (AA), carbohydrate esterase (CE), and carbohydrate-binding module (CBM) families.

To characterize the proteolytic potential of *K*. *sacchari* TAM1, predicted protein sequences were searched against a locally built MEROPS database (Release 12.5) using the command-line BLASTP tool (version 2.12.0) [31]. Significant hits were filtered based on an *E*-value cutoff of 10^−5^. Each identified sequence was assigned to a functional family and catalytic type according to the highest-scoring MEROPS match. The abundance and distribution of these families were further quantified to evaluate their potential roles in host tissue degradation.

To evaluate the potential for extracellular secretion, the 157 high-confidence protease sequences (identified via BLASTp against the MEROPS database with *E*-value 10^−5^) were subjected to secretome analysis. The presence and type of signal peptides were predicted using SignalP 6.0 (https://services.healthtech.dtu.dk/services/SignalP-6.0/ (accessed on 14 March 2026)). The ‘Other’ organism group was selected to accommodate the bacterial secretory system, and the ‘Slow’ mode was employed to ensure maximal predictive accuracy.

## 3. Results

### 3.1. Acaricidal Efficacy of Strain TAM1

#### 3.1.1. Comparative Acaricidal Efficacy

The acaricidal potential of strain TAM1 was evaluated through laboratory bioassays, with observed mortality recorded at 24 and 48 h post-treatment (Table 1). The data revealed distinct temporal patterns in efficacy among the treatments. At 24 h, the commercial acaricide abamectin exhibited a rapid effect, achieving 100% mortality. While the mortality rates for TAM1-TSB (8.3%) and TAM1-NB (5.0%) were relatively low at this early stage, they were significantly higher than those of the negative controls (*p* < 0.05). In contrast, Etoxazole (6.7%) showed no significant difference from the negative controls at 24 h (*p* > 0.05).

A substantial increase in mortality was observed in the TAM1 treatments between 24 and 48 h. By 48 h, the TAM1-TSB group reached 92.5% mortality, which was statistically comparable to Abamectin (100.0%, *p* > 0.05). The TAM1-NB treatment yielded 70.8% mortality at 48 h, significantly outperforming Etoxazole (18.3%). Throughout the experiment, mortality in all negative controls remained below 6%. Statistical analysis confirmed significant treatment effects at both 24 h (*χ*^2^ = 27.79, *df* = 6, *p* < 0.001) and 48 h (*χ*^2^ = 37.50, *df* = 6, *p* < 0.0001).

**Table 1:** Mortality of *Tetranychus urticae* adults after infection with *Kosakonia sacchari* strain TAM1, compared with the acaricides abamectin and etoxazole.

Treatment	Mortality (%) (Mean ± SD)	
	24 h	48 h
Control (water)	0.0 ± 0.0 c	2.5 ± 2.7 c
Control (Nutrient Broth)	1.7 ± 2.6 c	5.8 ± 3.8 c
Control (Tryptic Soy Broth)	2.5 ± 2.7 c	4.2 ±2.0 c
TAM1 in Nutrient Broth	5.0 ± 4.5 ab	70.8 ± 11.6 ab
TAM1 in Tryptic Soy Broth	8.3 ± 6.8 a	92.5 ± 5.2 a
Abamectin 10 μg/mL	100.0 ± 0.0 a	100.0 ± 0.0 a
Etoxazole 25 μg/mL	6.7 ± 2.6 bc	18.3 ± 9.3 bc

Twenty adult female mites were used per treatment, with six replicates. Means within a column followed by the same lowercase letter are not significantly different according to the Kruskal–Wallis test followed by Dunn’s post hoc test (*p* < 0.05, Bonferroni adjusted). Summary of Kruskal–Wallis tests: 24 h (*χ*^2^ = 27.79, *df* = 6, *p* < 0.001); 48 h (*χ*^2^ =37.50, *df* = 6, *p* < 0.0001).

#### 3.1.2. Dose–Response and Lethal Potency

To determine the lethal thresholds of TAM1, mites were exposed to a series of bacterial concentrations ranging from 10^1^ to 10^8^ CFU mL^−1^. The bioassays revealed that strain TAM1 exhibits potent, concentration-dependent acaricidal activity. Based on this observed dose–response relationship, the lethal concentrations (LC_50_ and LC_90_) and Probit regression parameters were estimated, as summarized in Table 2. Before conducting the formal Probit analysis, mortality data were pooled and corrected for the 8.0% control mortality using Abbott’s formula.

After correction, the highest concentration (10^8^ CFU mL^−1^) resulted in a corrected mortality of 92.4%. To account for the inherent biological variability within the bioassay and to provide a more robust estimation, a heterogeneity factor (*h* = *x*^2^/df = 9.55) was applied to adjust the 95% confidence intervals (CI). Notably, a plateau in mortality was observed at the lowest tested concentrations (10^1^ and 10^2^ CFU mL^−1^), which suggests a minimum threshold requirement for effective bacterial colonization (see Discussion for details).”

Based on this adjusted model, the estimated LC_50_ was 2.94 × 10^6^ CFU mL^−1^ (95%CI: 1.77 × 10^5^–4.88 × 10^7^), and the LC_90_ was 5.76 × 10^10^ CFU mL^−1^. (95% CI: 6.47 × 10^7^–5.13 × 10^13^) (Table 2). Notably, while the LC_90_ was estimated using the high mortality observed at 10^8^ CFU mL^−1^, the wide confidence interval suggests that this value represents a statistical extrapolation and should be interpreted as a theoretical threshold for near-complete mortality. The relationship between Strain TAM1 concentration and mite mortality was defined by the Probit regression equation:

*y* = 0.2986 x − 1.9314

where *y* is the probit of mortality and *x* is the log_10_-transformed concentration. The regression slope was 0.2986 ± 0.0241. Despite the statistically significant chi-square value (χ^2^ = 57.3), the model provides a conservative and representative assessment of the strain’s acaricidal potency.

### 3.2. Microscopy Observations of Strain TAM1 and Mites

**Figure 1:**
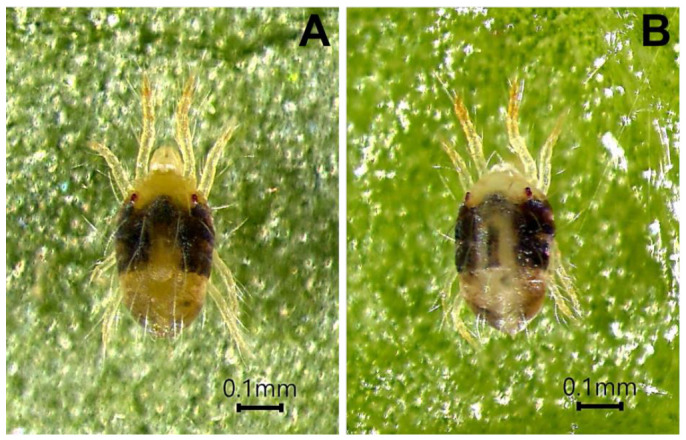
*Tetranychus urticae* adults 48 h after treated with *Kosakonia sacchari* strain TAM1. The infected mite (**A**) exhibits mortality and body darkening, compared with a healthy control mite (**B**). Scale bar = 0.1 mm.

Mites treated with strain TAM1 exhibited symptomatic body darkening and reduced mobility prior to death (Figure 1), contrasting with the active and translucent individuals in control groups. These visual signs represent physiological decline following treatment rather than confirmed internal colonization.

**Figure 2:**
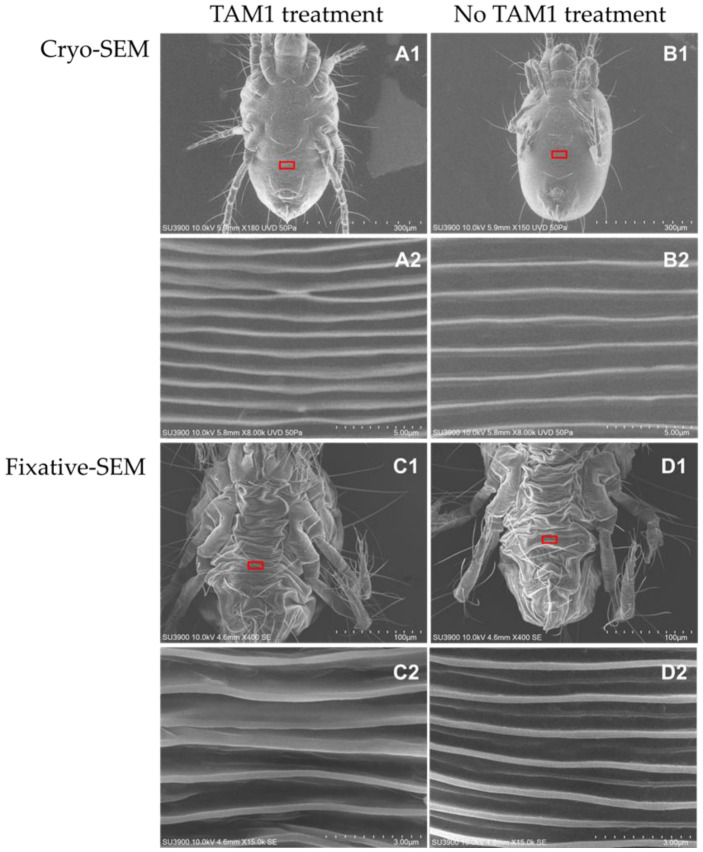
Scanning electron microscopy (SEM) images of the ventral abdominal opisthosoma of *Tetranychus urticae* 24 h after treatment with *Kosakonia sacchari* TAM1. (**A1**,**A2**) TAM1-treated mites (Cryo-SEM); (**B1**,**B2**) control mites (Cryo-SEM); (**C1**,**C2**) TAM1-treated mites (Fixative-SEM); (**D1**,**D2**) control mites (Fixative-SEM). Panels (**A1**–**D1)** show whole mites; the regions indicated by the red boxes are enlarged in (**A2**–**D2**), respectively.

**Figure 3:**
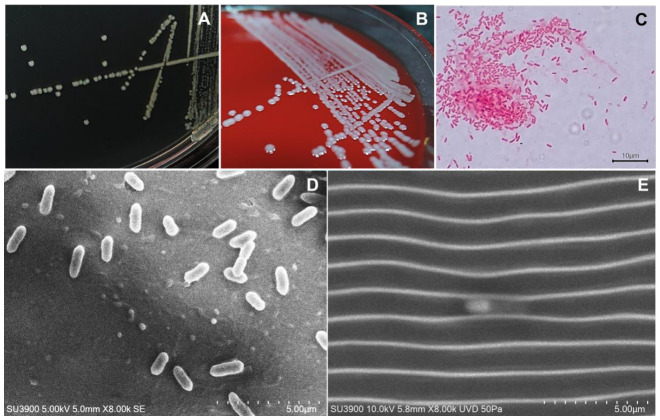
Colony morphology and cellular characteristics of the acaricidal *Kosakonia sacchari* strain TAM1. (**A**) Colonies on nutrient agar. (**B**) Colonies on hemolysin agar. (**C**) Gram-stained cells observed under a light microscope. (**D**) Scanning electron micrograph (SEM) showing the rod-shaped cellular morphology of strain TAM1. (**E**) Cryo-SEM examination of TAM1 cells securely attached between the ventral abdominal crest lines of *Tetranychus urticae* at 4 h post-treatment.

Ultrastructural examination revealed a distinct temporal progression in the interaction between strain TAM1 and *T. urticae*. By 24 h post-treatment, bacterial cells adhering to the external cuticle became remarkably scarce, even though pronounced localized structural disruption was observed on the ventral abdominal surface (Figure 2). Specifically, these localized morphological alterations, characterized by disrupted and irregular crest-line patterns, were consistently observed under both Cryo-SEM (Figure 2(A1,A2)) and conventional Fixative-SEM (Figure 2(C1,C2)). In the earlier stage of 4 h post-treatment, TAM1 cells were observed adhering to the ventral abdominal surface, specifically localized within the spaces between the cuticular crest lines (Figure 3E). These observations were consistent across multiple replicates (with localized damage observed in >80% of *n* = 10 examined specimens), confirming that the alterations were a direct result of TAM1 exposure rather than stochastic preparation artifacts.

In contrast, water-treated control mites maintained well-defined, intact, and regularly arranged crest lines across the same regions (Figure 2(B1,B2,D1,D2)). While the fixation and dehydration process in conventional SEM caused generalized body shrinkage in all specimens, the specific disruption of crest-line architecture was uniquely present in the TAM1-treated group across both preparation methods. No significant morphological deviations were identified in other body regions, indicating that the observed cuticular irregularities are associated with TAM1 infection rather than stochastic preparation artifacts.

### 3.3. Morphological and Physiological Characteristics of Strain TAM1

On NA medium, TAM1 colonies were circular, raised, with entire margins, and grey in color (Figure 3A). Colony morphology on blood agar was similar, and no hemolysis was observed (Figure 3B). Cells were Gram-negative, rod-shaped, and measured 1.5–1.9 × 0.4–0.7 µm, as observed by light microscopy (Figure 3C) and SEM (Figure 3D,E). These morphological traits are consistent with the characteristics of *K*. *sacchari*, as identified through the 16S rRNA and whole-genome analyses described below.

### 3.4. Growth Features of TAM1

**Figure 4:**
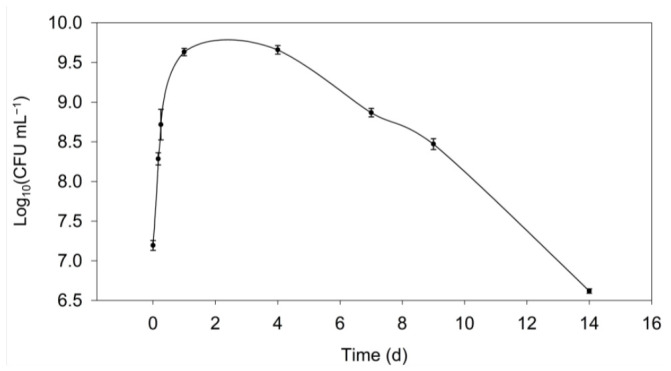
Growth curve of *Kosakonia sacchari* strain TAM1 at 30 °C. Viable cell density, expressed as colony-forming units (CFU), increased during the first 2–6 h (exponential phase) with an estimated doubling time of approximately 70.2 min. Cell density peaked between days 1 and 4 (stationary phase) and declined thereafter. Data are presented as means ± SD from three independent replicates.

The growth of *K*. *sacchari* strain TAM1 was monitored by measuring viable cell density as CFU at 30 °C. Rapid cell proliferation was observed during the first 6 h, corresponding to the exponential growth phase, with an estimated doubling time of approximately 70.2 min. Cell density increased from 1.6 × 10^7^ CFU mL^−1^ at the start of the experiment to 5.5 × 10^8^ CFU mL^−1^ by 6 h. The culture subsequently reached a stationary phase between days 1 and 4, with cell densities peaking at approximately 4.5 × 10^9^ CFU mL^−1^, followed by a gradual decline thereafter (Figure 4).

### 3.5. Temperature-Dependent Growth Profiles of Strain TAM1

**Figure 5:**
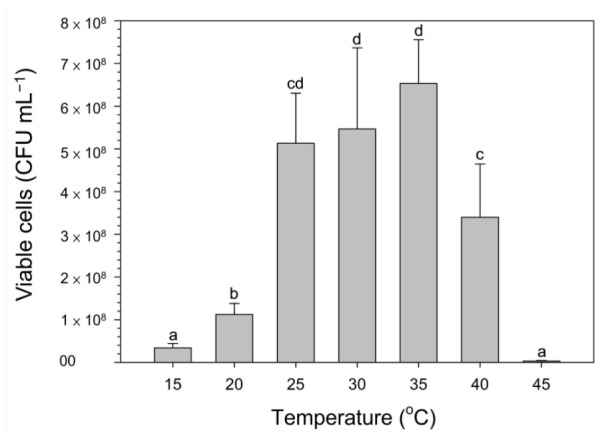
Effect of temperature on the growth of *Kosakonia sacchari* strain TAM1. Cells were cultured in TSB medium at different temperatures for 4 h, and bacterial growth was quantified based on viable counts (CFU mL^−1^). Values represent the mean ± standard deviation (SD) of three independent experiments. Different letters indicate significant differences among temperatures based on one-way ANOVA followed by Fisher’s LSD test (*p* < 0.05).

The growth of strain TAM1 was evaluated across a temperature range of 15–45 °C (Figure 5). One-way analysis of variance (ANOVA) followed by Fisher’s least significant difference (LSD) test showed that temperature significantly affected bacterial growth (*p* < 0.05). Bacterial growth showed a step-wise increase from 15 °C (group a) to 20 °C (group b), entering a high-growth plateau starting at 25 °C (group cd). Within this optimal range (25–35 °C), cell densities reached their highest levels (5.13 to 6.53 × 10^8^ CFU mL^−1^), with no significant difference observed between 25 °C and the numerical peaks at 30–35 °C (group d). As temperatures increased further, growth declined significantly at 40 °C (group c) and 45 °C (group a). The lowest growth levels were recorded at the extremes (15 °C and 45 °C), which showed no statistical difference from each other.

### 3.6. Chitinase and Gelatinase Activity

After 16 h of incubation, the TAM1 bacterial culture reached a density of 13.14 ± 0.13 × 10^9^ CFU mL^−1^. Chitinase and gelatinase activities were subsequently determined in both the culture supernatant and the cell lysate.

For chitinase activity, no detectable levels were observed in the culture supernatant, whereas the cell lysate exhibited significant enzymatic activity. The specific activities of the three chitinolytic enzymes—β-N-acetylglucosaminidase, chitobiosidase, and endochitinase—were 15.31, 3.10, and 0.88 nmol p-nitrophenol min^−1^ per 10^9^ CFU, respectively (Table 3). Among these, β-N-acetylglucosaminidase showed the highest activity, followed by chitobiosidase and endochitinase.

Similarly, the gelatinase activity of strain TAM1 was 0.30 µmol L-leucine equivalents per 10^9^ cells in the culture supernatant, while the cell lysate exhibited a markedly higher activity of 12.63 µmol L-leucine equivalents per 10^9^ cells (Table 3).

Overall, both chitinase and gelatinase activities were predominantly detected in the cell lysate rather than in the culture supernatant. These results explicitly demonstrate that, under the tested in vitro conditions, these enzymes are primarily localized within the cellular fraction (such as the cytoplasm or periplasm) or remain cell-bound. The lack of significant extracellular activity suggests that constitutive secretion of these enzymes into the surrounding environment does not occur in standard laboratory media, indicating that their release might require specific host-related cues or alternative delivery mechanisms during the infection process.

### 3.7. 16S rRNA Gene

Sequencing of the cloned 16S rRNA gene yielded a nearly complete 1495 bp sequence, which was deposited in GenBank under accession number PX048888. This sequence was highly consistent with the 1528 bp 16S rRNA sequence derived from whole-genome sequencing mentioned below. BLAST analysis revealed 99.87% identity with *Kosakonia sacchari* DSM107661 (CP040677.1) Other close matches included several *Kosakonia* spp. and *Enterobacter* sp. (>99.26% similarity), while *Klebsiella quasipneumoniae* strains showed >98.60% similarity (Table 4).

### 3.8. Whole Genome Sequencing and Functional Annotation

#### 3.8.1. Genome Assembly and General Features

Illumina MiSeq sequencing generated 4,430,440 paired-end reads (301 bp), yielding 1,333,562,440 bp of data. Nanopore sequencing produced 590,008 long reads totaling 7,540,018,691 bp, with a maximum read length of 149,150 bp (mean 12,779 bp). Hybrid assembly resulted in two circular contigs: Contig 1 (chromosome) of 5,066,903 bp (183× coverage) containing 4860 protein-coding genes, 78 tRNA genes, and 22 rRNA genes; and Contig 2 (plasmid) of 164,574 bp (305× coverage) containing 166 protein-coding genes and 5 tRNA genes but no rRNA genes (Table 5). The complete sequences were deposited in GenBank under BioProject PRJNA946387 (BioSample SAMN33823087), with accession numbers CP120954 (chromosome) and CP120955 (plasmid). Raw sequencing reads have been deposited in the Sequence Read Archive (SRA) under the study accession SRP428848.

In the genome of TAM1, the chromosome encodes chitin deacetylase, gelatinase, and related proteases, as well as multiple type II toxin–antitoxin (TA) system components, including RelB/DinJ and HicB family antitoxins. The plasmid carries genes encoding ParD family antitoxins, RelE/ParE family toxins, and VapC family toxins. Among these, the activities of chitinase and gelatinase have been experimentally confirmed in this study, while the presence of the other genes provides a genomic basis for TAM1’s bioactivity, although their specific functions require further experimental validation.

#### 3.8.2. Genomic-Based Taxonomic Placement and ANI Analysis

ANI comparisons supported the 16S rRNA results, placing TAM1 closest to *K*. *sacchari* DSM107661 (ANI = 98.67%) and *Enterobacter* sp. R4-368 (ANI = 98.60%) (Table 4). Other species showed ANI values <95%. Based on these results, TAM1 was tentatively assigned to *K*. *sacchari* for sequence submission, although it had an ANI of 98.60 to *Enterobacter* sp. R4-368 (Table 4).

#### 3.8.3. Genomic Basis of Acaricidal Potential: Chitin-Degrading Repertoire

Whole-genome analysis of *K*. *sacchari* strain TAM1 provided insights into study potential acaricidal mechanisms. Current annotation revealed two putative chitin-associated genes that may contribute to mite lethality. The first gene, TAM1_4753 (759 bp), was predicted to encode a chitin disaccharide deacetylase (CDD, 252 amino acids; pfam04794, COG3394). The second, TAM1_1127 (555 bp), encoded a chitin-binding protein (CBP, 184 amino acids; pfam 03067, COG3397).

BLASTn analysis showed that CDD gene shared >97.50% sequence identity with homologues from *K*. *sacchari* DSM107661 and *Enterobacter* sp. R4-368, but ≤93.54% identity with those of other bacterial taxa (Table 6). In contrast, CBP gene exhibited 99.28% identity with *K*. *sacchari* DSM107661, 98.92% with *Enterobacter* sp. R4-368, and no hits to other *Kosakonia* strains. The next hits ≤72.19% to strains of *Providencia* in Morganellaceae. Notably, no acaricidal activity has been previously reported from either *K*. *sacchari* or *Enterobacter* sp. R4-368.

The identification of these putative chitin-associated genes aligns with the chitinase and gelatinase activities detected in the preliminary enzymatic assays (Table 3). To further characterize the molecular basis of these activities, a refined functional profiling was performed via the CAZy database to systematically reconcile genomic evidence with the observed chitinolytic potential. Functional profiling via the CAZy database confirmed a sophisticated hydrolytic repertoire, including the primary chitinase XGA90010.1 (GH18), and two β-N-acetylhexosaminidases, XGA91119.1 and XGA87419.1 (GH20). Structural modification and auxiliary activity genes were characterized as a CE4 family chitin deacetylase (XGA87084.1) and an AA10 family lytic polysaccharide monooxygenase (LPMO, XGA90006.1). Furthermore, the TAM1 genome encodes multiple substrate-binding modules, such as the unique architecture of XGA87092.1 (MltD), which integrates a GH23 domain with tandem repeats of the CBM50 module. Refined annotation further suggests that TAM1_4753 (Pfam 04794) is putatively identified as a chitin disaccharide deacetylase, while TAM1_1127 (Pfam 03067) is predicted to encode a chitin-binding protein harboring a LysM (CBM50) domain (Table 7).

#### 3.8.4. Profiling of the Proteolytic System and Secretory Potential

**Figure 6:**
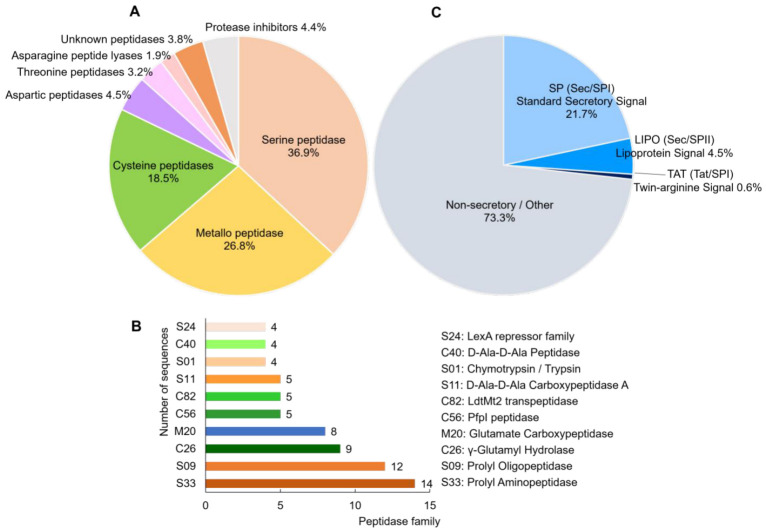
Comprehensive analysis of the proteolytic system in *Kosakonia* sacchari TAM1. (**A**) Distribution of peptidase catalytic types based on MEROPS classification. (**B**) Abundance of the top 10 most frequent peptidase families. (**C**) Prediction of secretory pathways and signal peptide types for the 157 identified peptidase sequences using SignalP-6.0.

In addition to chitinolytic enzymes, the proteolytic system of TAM1 was comprehensively characterized. To elucidate the genetic basis of the observed proteolytic activities, the complete protease repertoire was identified by searching the predicted proteome against the MEROPS database. A total of 157 high-confidence protease-encoding genes were annotated (*E*-value ≤ 10^−5^) (Table S1). The proteolytic landscape of TAM1 (Figure 6A) is dominated by serine peptidases (36.9%, 58 genes), metallopeptidases (26.8%, 42 genes), and cysteine peptidases (18.5%, 29 genes). Furthermore, the genome encodes a diverse array of minor catalytic types, including aspartic (4.5%, 7 genes), threonine (3.2%, 5 genes), and asparagine peptidases (1.9%, 3 genes). Notably, unassigned peptidases (including U32 and U69 families; 3.8%, 6 genes) and protease inhibitors (4.4%, 7 genes) were also identified, suggesting sophisticated regulatory mechanisms for protein degradation.

The high prevalence of metallopeptidases aligns consistent with the significant gelatinase activity detected in the TAM1 lysate. Among the top 10 most expanded families, S33 (14 genes), S09 (12 genes), and M20 (8 genes) were the most prevalent (Figure 6B).

To evaluate the secretory potential of the identified protease repertoire, the 157 protein sequences were analyzed for N-terminal signal peptides (SPs) using SignalP 6.0 (Table S1). The analysis predicted that 42 sequences (26.8%) contain recognizable secretory signals, suggesting they are targeted for translocation across the cytoplasmic membrane. The majority of these predicted secretome components utilize the Sec-dependent pathway, with 34 sequences classified as containing standard secretory signals (Sec/SPI). Furthermore, 7 sequences were identified as lipoproteins (Sec/SPII), characterized by the presence of a conserved lipobox motif for membrane anchoring.

In contrast, only a single sequence, predicted as an α/β hydrolase (XGA88889.1), was predicted to be transported via the Twin-arginine translocation (Tat/SPI) pathway with a high probability (1.000). The remaining 115 sequences were categorized as “Other,” indicating a lack of detectable N-terminal signal peptides (Figure 6C). Notably, the reliance on a single Tat-routed sequence (XGA88889.1) highlights the dominance of the Sec-dependent system for protease secretion in this strain.

## 4. Discussion

The development of microbial pesticides with diverse acaricidal mechanisms offers a sustainable strategy to mitigate chemical resistance in *T*. *urticae*. Strain TAM1, isolated from mite cadavers in Taiwan, demonstrates potent acaricidal activity. Our results show that TAM1 achieves over 90% mortality within 48 h, demonstrating a level of efficacy comparable to the commercially established fermentation product abamectin [32]. Furthermore, TAM1 outperformed the synthetic acaricide etoxazole, which primarily targets immature stages via the inhibition of chitin biosynthesis [33].

Regarding the dose–response dynamics, the mortality plateau observed at sub-lethal concentrations (19.0% and 17.0% at 10^1^ and 10^2^ CFU mL^−1^, respectively) likely reflects a concentration threshold required for effective infection by *K. sacchari* TAM1. At these minimal initial titers, the bacterial load may be insufficient to reach the quorum required for coordinated virulence factor secretion, or it may be effectively managed by the basal innate immune defenses of *T. urticae*. It is only when the concentration exceeds this establishment threshold that a consistent, dose-dependent pathogenic cascade is triggered, highlighting that the interaction between TAM1 and the host mite involves a concentration-dependent establishment phase rather than a simple linear toxic effect from the lowest doses.

The superior performance of TAM1 grown in TSB compared to NB suggests that medium composition significantly influences the production of its bioactive compounds. TAM1 exhibited optimal growth between 25 and 35 °C, which is consistent with the characteristics of other effective biocontrol agents like *Bacillus subtilis* and *Pseudomonas fluorescens* [34,35]. This broad thermal tolerance, coupled with the ability to maintain high cell densities, is highly advantageous for industrial-scale fermentation [36]. The subsequent decline in viable counts after four days likely results from nutrient exhaustion and byproduct accumulation, which is a standard physiological observation in batch cultures once they surpass the exponential growth phase [37,38].

Experimental assays detected significant chitinase and gelatinase activities in TAM1. Based on the framework by Poria et al. [39], β-N-acetylglucosaminidase and chitobiosidase are regarded as exo-chitinases that sequentially release N-acetylglucosamine or chitobiose units from the non-reducing ends of chitin polymers. Endochitinase represents the endo-acting type that randomly cleaves internal β-1,4-glycosidic linkages within the chitin chain.

The enzymatic assays of *K*. *sacchari* TAM1 revealed significant levels of β-N-acetylglucosaminidase, chitobiosidase, endochitinase, and gelatinase activities, which are highly consistent with the identification of a core chitinolytic and proteolytic repertoire within its genome. The chitinolytic system includes the primary GH18 chitinase (XGA90010.1) and terminal hexosaminidases from the GH20 family (XGA91119.1 and XGA87419.1). Specifically, the high β-N-acetylglucosaminidase activity correlates with the expansion of GH20 family genes, which encode enzymes essential for the hydrolysis of chitin-derived oligomers into monomers. Furthermore, the genomic characterization of auxiliary elements, such as the AA10 family LPMO (XGA90006.1) and CE4 family chitin deacetylases (TAM1_4753), provides a genetic basis for the multi-step degradation process observed in the enzymatic profiles [39].

Notably, while these degradative activities were prominent within the cell lysate, they remained undetectable in the culture supernatant. We explicitly acknowledge that these current in vitro data do not demonstrate extracellular secretion under the tested laboratory conditions. This intracellular localization is initially inconsistent with a model of constitutive external cuticle digestion, as the enzymes were not observed to be released into the surrounding medium. Consequently, all secretion-related claims derived from our genomic analysis remain predicted bioinformatic secretomes rather than experimentally demonstrated secretory events.

In many entomopathogenic bacteria, the production and release of hydrolytic enzymes are specifically triggered by surface-sensing mechanisms or host-derived chemical cues encountered during contact with the host cuticle [40,41]. Such regulated delivery—or alternative mechanisms such as contact-dependent systems or the release of the intracellular arsenal upon localized cell lysis on the cuticle surface—may explain the observed pathological outcomes despite the lack of constitutive secretion in laboratory media. At present, external enzymatic degradation remains a hypothesis requiring further experimental validation, such as proteomic analysis of the supernatant under host-inducing conditions to confirm active secretion pathways.

Based on observations of other acaricidal bacteria, Al-Azzazy et al. [13] hypothesized that high efficacy of certain strains against small-bodied mites could be attributed to superior colonizing capacity and strong cuticular adhesion. However, our observations—using both conventional SEM and Cryo-SEM—revealed a relative scarcity of bacterial cells adhering to the mite cuticle at 24 h post-treatment. This temporal disconnect—where bacteria are present at 4 h but scarce by 24 h while structural damage manifests later—suggests that the primary bacterial-mediated compromise occurs during an early window of exposure.

Furthermore, the interpretation of the “bacterial-mediated structural damage” observed at 24 h should be qualified. It is possible that the initial enzymatic weakening of the cuticle by TAM1 accentuates secondary processes, such as host autolytic activities or dehydration artifacts during SEM sample preparation. Therefore, the severe morphological alterations likely represent a cumulative pathological outcome initiated by TAM1 rather than continuous direct degradation.

Rather than relying on persistent, high-density colonization, TAM1 is hypothesized to employ a targeted approach where physical anchoring—potentially facilitated by identified CBM50 modules—concentrates proteolytic and chitinolytic activities directly on the host cuticle. This requirement for direct proximity is supported by our finding that enzymes are predominantly cell-associated (lysate). It is plausible that even transient attachment or a small pioneer population is sufficient to initiate the degradation of the host’s physical barrier, with the 24 h SEM sampling capturing the pathological outcome rather than the peak phase of attachment.

The efficacy of this targeted approach is further explained by the molecular composition of the host’s physical barrier. Recent transcriptomic analysis of *T. urticae* has identified 59 genes encoding cuticular proteins (CPs) that are actively involved in the structural formation of the mite’s exoskeleton [29]. These CPs, particularly those within the CPR family, are characterized by conserved domains rich in proline and alanine residues, which are essential for maintaining the mechanical stability of the chitin-protein matrix [42]. The identification of specialized S8 (serine peptidase) and M4 (metallopeptidase) peptidases in the TAM1 genome—which exhibit significant gelatinase-like activity toward proline-rich substrates—provides a plausible molecular basis for the localized cuticular disruption observed.

The alignment of microscopic, enzymatic, and genomic data suggests that TAM1 utilizes a synergistic, multi-target mode of action. Beyond the identified hydrolases, the possibility that specific cell-surface-associated factors or membrane-integrated components contribute directly to host mortality during the initial contact phase offers a compelling direction for future functional validation. Such a mechanism would be consistent with the rapid mortality and severe ultrastructural damage observed despite the sparse bacterial presence at the 24 h mark.

Additionally, genome analysis identified elements such as toxin–antitoxin (TA) systems and membrane-associated proteins that may further bolster this bioactivity. The TAM1 chromosome encodes various type II TA system components, including RelB/DinJ and HicB family antitoxins, while the plasmid carries RelE/ParE and VapC family toxins. These TA systems may regulate secondary metabolism and enhance TAM1 persistence under environmental stress [43]. Similarly, membrane-associated porins or lipid-trapping mechanisms have been linked to mite mortality in other species like *Photorhabdus luminescens* strain 0805-P2R [17,44]. While these genes require further functional validation, they provide a plausible genomic basis for the strain’s potent acaricidal potential when working alongside the confirmed chitin-protein degrading machinery.

Conventional SEM can induce minor artifacts in soft-bodied arthropods due to chemical fixation, as seen in the slight surface irregularities of our control mites (Figure 2(D1,D2)) compared to the well-preserved structures in Cryo-SEM (Figure 2(B1,B2)). However, the pronounced structural alterations observed in TAM1-treated mites (Figure 2(A1,A2,C1,C2) point toward bacterial-mediated cuticular compromise that exceeds these fixation effects. We hypothesize that the identified chitinolytic and gelatinolytic enzymes contribute to this degradation of the host’s physical barrier, potentially facilitating further bioactivity [45]. The alignment of microscopic, enzymatic, and genomic data suggests that TAM1 utilizes a synergistic, multi-target mode of action rather than relying on a single mechanism.

Average Nucleotide Identity (ANI) values exceeding the 95–96% threshold typically indicate that two strains belong to the same species [46]. In this study, TAM1 was tentatively assigned to *K*. *sacchari* based on an ANI similarity of 98.67% to the strain DSM 107661. It should be noted that strain DSM 107661 is a collection in Leibniz Institute DSMZ-German Collection of Microorganisms and Cell Cultures. Description of the strain is currently limited. Based on metadata in the genome sequence (CP040677.1), the bacterial strain was isolated from the rhizosphere soil/topsoil of a rice field in Sri Lanka in 2014. The strain also shares 98.60% similarity with *Enterobacter* sp. R4-368, a strain with an uncertain species status. In contrast, three *K*. *sacchari* strains including Type strain SP1^T^ show ANI values <95% (Table 4). Although a comprehensive taxonomic re-evaluation is beyond the scope of this study, our analysis highlights a complex phylogenetic structure within the *K. sacchari* group that warrants further investigation. Consequently, the assignment of TAM1 to *K*. *sacchari* is considered probable and serves as a matter of practical convenience for presenting our findings on its biocontrol potential, but it must be confirmed by dedicated taxonomic studies in the future.

Despite the potent acaricidal activity demonstrated by strain TAM1, several limitations of this study should be considered. First, our results are based on controlled laboratory bioassays; the efficacy and stability of TAM1 under complex field conditions—where variables such as UV radiation and humidity fluctuations are present—remain to be validated. Second, while genome analysis identified multiple toxin–antitoxin (TA) systems, their functional involvement in host–pathogen interactions is currently unverified. These systems may primarily facilitate bacterial persistence under environmental stress rather than acting as primary virulence factors.

Finally, we acknowledge certain discrepancies between the genomic repertoire and the observed enzymatic profiles. The presence of specific hydrolase genes does not always guarantee detectable activity in vitro, suggesting that these enzymes may require specific host-derived cues for their expression and secretion. Addressing these gaps through functional genomics and field-scale investigations will be essential to fully evaluate the biocontrol potential of TAM1.

## 5. Conclusions

Strain TAM1 (*K*. *sacchari*), isolated from a deceased mite in Taiwan, is a highly effective acaricidal agent. It achieves >90% adult mortality in *T*. *urticae* within 48 h, characterized by distinct physical symptoms such as body darkening and abdominal crest deformation. The integration of phenotypic evidence, acaricidal enzymatic assays, and whole genome sequence analysis confirms that TAM1 operates through complex, multi-target mechanisms involving both chitinase and gelatinase activities. These findings position TAM1 as a promising candidate for sustainable integrated pest management (IPM) and acaricide resistance mitigation.

However, this study represents a foundational “proof-of-concept” phase. Future research will evaluate the environmental safety and specificity of TAM1 toward non-target and beneficial organisms, including predatory mites. Additionally, animal safety trials will be conducted in subsequent stages of evaluation. Furthermore, investigations will focus on transitioning from laboratory efficacy to semi-field and field trials, alongside the development of stable formulations to ensure bacterial survival and consistent performance under fluctuating environmental conditions.

## Figures and Tables

**Table 2:** Lethal concentrations (LC) of strain TAM1 against adult female *Tetranychus urticae* (*n* = 100) determined by Probit analysis.

Treatment	LC_50_ (95% CI) ^a^	LC_90_ (95% CI) ^a^	Slope ± SE	χ^2^ (df)	*p*-Value
Strain TAM1	2.94 × 10^6^	5.76 × 10^10^	0.2986 ± 0.0241	57.3 (6)	<0.0001
	(1.77 × 10^5^–4.88 × 10^7^)	(6.47 × 10^7^–5.13 × 10^13^)			

^a^ Lethal concentrations are expressed in CFU mL^−1^. Mortality data were corrected using Abbott’s formula (control mortality = 8.0%) prior to Probit regression. As the Pearson chi-square test was significant (*p* < 0.05), a heterogeneity factor (*h* = *x*^2^/df = 9.55) was applied to adjust the 95% confidence intervals (CI), ensuring a conservative estimation of the lethal range. The inclusion of the 10^8^ CFU mL^−1^ treatment, which achieved 92.4% corrected mortality, provided the empirical threshold required for LC_90_ estimation.

**Table 3:** Chitinase and gelatinase activities of *Kosakonia sacchari* strain TAM1 in culture supernatant and cell lysate.

Sample Fraction	Chitinase Activity (U per 10^9^ CFU)			Gelatinase Activity (U per 10^9^ CFU)
	β-N-Actylglucosaminidase	Chitobiosidase	Endochitinase	
Culture supernatant	not detected	not detected	not detected	0.30 ± 0.04
Cell lysate	15.31 ± 2.07	3.10 ± 0.30	0.88 ± 0.13	12.63 ± 1.21

Note: Values represent the mean ± standard deviation (*n* = 3). Enzyme activities were normalized to cell density (10 ^9^ CFU) to provide a per-cell enzymatic potential consistent with the dosages used in the bioassays. Unit definitions: One unit of chitinase activity releases 1.0 nmol of p-nitrophenol from the appropriate substrate per minute at 37 °C. One unit of gelatinase activity releases 1.0 µmol of L-leucine from the appropriate substrate per minute at 37 °C.

**Table 4:** Taxonomic evaluation of the *Kosakonia sacchari* strain TAM1 based on 16S rRNA gene sequence similarity (BLAST search against GenBank nr database) and whole-genome average nucleotide identities (ANIs).

Bacterial Name	GenBank	Geographic	16S rRNA Gene		Whole Genome	
	Accession	Origin	Similarity (%)	Rank	ANI (%)	Rank
*Kosakonia sacchari* DSM107661	CP040677.1	Sri Lanka	99.87	1	98.67	1
*Kosakonia sacchari* SP1^T^	CP007215.3	China	99.73	2	94.59	5
*Kosakonia sacchari* KS2022	CP137744.1	China	99.60	3	94.67	3
*Enterobacter* sp. R4-368	CP005991.1	Singapore	99.60	3	98.60	2
*Kosakonia pseudosacchari* BDA62-3	CP063425.1	Italy	99.40	5	94.04	6
*Kosakonia sacchari* BO1	CP016337.1	Japan	99.26	6	94.59	4
*Kosakonia pseudosacchari* RX.G5M8	CP115712.1	Hong Kong	99.20	7	93.97	7
*Klebsiella quasipneumoniae* N18-04101	CP047281.1	Canada	98.93	8	81.32	9
*Klebsiella quasipneumoniae* isolate 0	OW968431.1	Spain	98.60	9	81.33	8
*Klebsiella quasipneumoniae* subsp. *similipneumoniae* 09A323	CP084783.1	Greece	98.60	9	81.28	10

**Table 5:** Summary of sequence data and assembly metrics for the *Kosakonia sacchari* strain TAM1.

Parameter	Value
Illumina short reads	4,430,440
	Total = 1,333,562,440 bp
	Each = 301 bp
Nanopore long reads	590,008
	Total length = 7,540,018,691 bp
	Read N50 length = 18,325 bp
	Mean read length = 12,779.5 bp
	Maximum read length = 149,150 bp
Assembly	
Contig number	2 (Chromosome and plasmid)
Chromosome	5,066,903 bp; GenBank accession: CP120954
Average coverage	183×
Open reading frames	4860
rRNA gene	22
5S rRNA genes	8
16S rRNA genes	7
23S rRNA genes	7
tRNA genes	78
GC content	53.9%
Plasmid	164,574 bp; GenBank accession: CP120955
Average coverage	305×
Open reading frame	166
rRNA genes	0
tRNA genes	5
GC content	53.9%

**Table 6:** Selected results from BLASTn searches against the NCBI nucleotide database using two chitinase genes (TAM1_4753 and TAM1_1127) from *Kosakonia sacchari* strain TAM1 as queries.

Species	Bit Score	Query Coverage (%)	Identity (%)	Accession
**Query: TAM1_4753 (759 bp, chitin disaccharide deacetylase)**				
*Enterobacter* sp. R4-368	1303	100	97.63	CP005991.1
*Kosakonia sacchari* DSM 107661	1297	100	97.50	CP040677.1
*Kosakonia sacchari* KS2022	1131	100	93.54	CP137744.1
*Kosakonia sacchari* BO-1	1098	100	92.76	CP016337.1
*Kosakonia sacchari* SP1^T^	1098	100	92.75	CP007215.3
*Kosakonia pseudosacchari* BDA62-3	1042	100	91.44	CP063425.1
*Kosakonia pseudosacchari* RX.G5M8	1020	100	90.91	CP115712.1
*Kosakonia radicincitans* GXGL-4A	477	99	78.46	CP015113.1
*Kosakonia radicincitans* DSM 107547	472	99	78.33	CP040392.1
*Kosakonia radicincitans* D4	466	99	78.20	LT799040.1
**Query: TAM1_1127 (555 bp, chitin-binding protein)**				
*Kosakonia sacchari* DSM 107661	984	100	99.28	CP040677.1
*Enterobacter* sp. R4-368	975	100	98.92	CP005991.1
*Providencia vermicola* P13	270	94	72.19	CP097327.1
*Providencia zhejiangensis* SKLX146130	270	94	72.19	CP169566.1
*Providencia vermicola* Z34CR2292	270	94	72.19	CP145938.1
*Providencia vermicola* PVA41	270	94	72.19	CP116222.1
*Providencia* sp. PROV080	266	94	72.00	CP096346.1
*Providencia* sp. 21OH12SH02B-Prov	266	94	72.00	CP114796.1
*Providencia stuartii* CMC-4104	266	94	72.00	CP095443.1
*Providencia stuartii* CAVP490	266	94	72.00	CP119546.1

**Table 7:** Correspondence of chitin-degrading genes and CAZy families identified in the TAM1 genome.

Gene ID/ Accession	Gene Location	Length (aa)	Family (Domain)	Predicted Protein Product
TAM1_4753	4,962,678–4,961,920	252	CE4, Pfam04794 (CDD)	Chitin disaccharide deacetylase
TAM1_1127	1,153,927–1,154,481	184	CBM50, pfam03067 (COG)	LPMO (GbpA-like Chitin-binding protein)
XGA90010.1	1,157,182–1,159,071	629	GH18	Chitinases
XGA91119.1	2,413,413–2,416,067	884	GH20	β-N-acetylhexosaminidase
XGA87419.1	3,319,049–3,321,433	794	GH20	β-N-acetylhexosaminidase
XGA87084.1	2,931,419–2,932,360	313	CE4	allantoinase PuuE
XGA90006.1	1,153,903–1,154,481	192	AA10	Lytic polysaccharide monooxygenase (N-acetylglucosamine-binding protein A)
XGA88742.1	4,821,840–4,822,574	244	CBM50	Amidase activator ActS
XGA91407.1	4,939,246–4,940,319	357	CBM50	Murein hydrolase activator NlpD
XGA89794.1	922,600–923,928	442	CBM50	Murein DD-endopeptidase MepM
XGA87092.1	2,938,540–2,939,910	456	CBM50 GH23	Membrane-bound lytic murein transglycosylase D (with tandem CBM50)
XGA89859.1	991,370–991,981	209	GH23	Membrane-bound lytic murein transglycosylase EmtA
XGA91399.1	4,756,025–4,757,107	360	GH23	Membrane-bound lytic murein transglycosylase MltC
XGA91224.1	217,167–218,759	530	GH23	Membrane-bound lytic murein transglycosylase MltF
XGA87270.1	3,158,649–3,160,589	646	GH23	Membrane-bound lytic murein transglycosylase SltY
XGA91318.1	2,644,135–2,644,578	147	GH24	Lysozyme (Endolysin/Autolysin)
XGA87396.1	3,294,381–3,294,833	150	GH24	Lysozyme (Endolysin/Autolysin)
XGA89135.1	163,461–163,970	169	GH24	Lysozyme (Endolysin/Autolysin)
XGA90617.1	1,862,070–1,862,510	146	GH24	Lysozyme (Endolysin/Autolysin)
XGA87393.1	3,292,421–3,292,876	151	GH24	Lysozyme (Endolysin/Autolysin)
XGA89875.1	1,008,946–1,009,779	277	GH24	Lysozyme (Endolysin/Autolysin)
XGA89075.1	112,737–113,252	171	GH24	Lysozyme (Endolysin/Autolysin)
XGA88653.1	4,716,666–4,719,716	1016	GH24	Lysozyme (Endolysin/Autolysin)

## Data Availability

The 16S rRNA gene sequence of strain TAM1 is available in GenBank under accession number PX048888. The complete genome and plasmid sequences have been deposited in GenBank under accession numbers CP120954 and CP120955, respectively. All genomic data are associated with BioProject PRJNA946387 and BioSample SAMN33823087. The raw sequencing reads (Illumina and Nanopore) have been deposited in the Sequence Read Archive (SRA) under study accession SRP428848.

## Supplementary Materials

The following supporting information can be downloaded at https://www.mdpi.com/article/10.3390/microorganisms14061192/s1, Table S1. Repertoire of peptidases and protease inhibitors, including signal peptide prediction, in *Kosakonia sacchari* TAM1. This table provides details on 157 high-confidence peptidase-encoding genes, including their MEROPS classification and N-terminal signal peptide analysis performed via SignalP 6.0.

## References

[1] Grbić M., Van Leeuwen T., Clark R.M., Rombauts S., Rouzé P., Grbić V., Osborne E.J., Dermauw W., Ngoc P.C.T., Ortego F. (2011). The genome of *Tetranychus urticae* reveals herbivorous pest adaptations. Nature.

[2] Ho C.C. (2000). Spider-mite problems and control in Taiwan. Exp. Appl. Acarol..

[3] Ho C.C., Lo K.C. (1979). Influence of temperature on life history and population parameters of *Tetranychus urticae*. J. Agric. Res. China.

[4] Lin M.Y. (2019). Control Efficiency of various miticides for *Tetranychus urticae* on Papaya. J. Plant. Med..

[5] Dermauw W., Wybouw N., Rombauts S., Menten B., Vontas J., Grbić M., Clark R.M., Feyereisen R., Van Leeuwen T. (2013). A link between host plant adaptation and pesticide resistance in the polyphagous spider mite *Tetranychus urticae*. Proc. Natl. Acad. Sci. USA.

[6] Mota-Sanchez D., Wise C.J. (2025). The Arthropod Pesticide Resistance Database.

[7] Tabashnik B.E. (1994). Evolution of resistance to *Bacillus thuringiensis*. Annu. Rev. Entomol..

[8] Jiang Y., Wang J. (2023). The registration situation and use of mycopesticides in the world. J. Fungi..

[9] Bugeme D.M., Knapp M., Boga H.I., Ekesi S., Maniania N.K. (2014). Susceptibility of developmental stages of *Tetranychus urticae* (Acari: Tetranychidae) to infection by *Beauveria bassiana* and *Metarhizium anisopliae* (Hypocreales: Clavicipitaceae). Int. J. Trop. Insect Sci..

[10] Lazo G.R., Chang S.C., Shih H.T., Huang H., Wallis C.M., de León A.Á.P., Chen J. (2025). Mini-review on microbial pesticide research for crop protection assisted by generative AI. J. Taiwan Agric. Res..

[11] Chang S.C., Lee C.C., Chiang M.Y. (2026). Research and development of pathogenic microorganisms for the control of major agricultural pests. J. Taiwan Agric. Res..

[12] Aksoy H.M., Ozman-Sullivan S.K., Ocal H., Celik N., Sullivan G.T. (2008). The effects of *Pseudomonas putida* biotype B on *Tetranychus urticae* (Acari: Tetranychidae). Exp. Appl. Acarol..

[13] Al-Azzazy M.M., Alsohim A.S., Yoder C.E. (2020). Biological effects of three bacterial species on *Tetranychus urticae* (Acari: Tetranychidae) infesting eggplant under laboratory and greenhouse conditions. Acarologia.

[14] Quessaoui R., Bouharroud R., Amarraque A., Ajerrar A., Mayad E.H., Chebli B., Dadi M., Elaini R., El Filali F., Walters A.S. (2017). Ecological applications of *Pseudomonas* as a biopesticide to control two-spotted mite *Tetranychus urticae*: Chitinase and HCN production. J. Plant Prot. Res..

[15] Li X.Y., Munir S., Cui W.Y., He P.J., Yang J., He P.F., Wu Y.X., Wang Y.H., He Y.Q. (2019). Genome sequence of *Bacillus velezensis* W1, a strain with strong acaricidal activity against two-spotted spider mite (*Tetranychus urticae*). Appl. Ecol. Environ. Res..

[16] Golec J.R., Hoge B., Walgenbach J.F. (2020). Effect of biopesticides on different *Tetranychus urticae* Koch (Acari: Tetranychidae) life stages. Crop Prot..

[17] Hsieh T.T., Chang J.C., Hsieh C., Tseng J.T., Lin S.J., Yang C.J., Hsieh F.C., Nai Y.S. (2023). Miticidal activity of *Photorhabdus luminescens* for controlling two spider mites, *Tetranychus urticae* and *Tetranychus kanzawai*, in *Carica papaya*. BioControl.

[18] Kämpfer P., McInroy J.A., Doijad S., Chakraborty T., Glaeser S.P. (2016). *Kosakonia pseudosacchari* sp. nov., an endophyte of *Zea mays*. Syst. Appl. Microbiol..

[19] Romano I., Ventorino V., Ambrosino P., Testa A., Chouyia F.E., Pepe O. (2020). Development and application of low-cost and eco-sustainable bio-stimulant containing a new plant growth-promoting strain *Kosakonia pseudosacchari* TL13. Front. Microbiol..

[20] Shahid M., Ameen F., Maheshwari H.S., Ahmed B., AlNadhari S., Khan M.S. (2021). Colonization of Vigna radiata by a halotolerant bacterium *Kosakonia sacchari* improves the ionic balance, stressor metabolites, antioxidant status and yield under NaCl stress. Appl. Soil Ecol..

[21] Van Leeuwen T., Van Pottelberge S., Tirry L. (2005). Comparative acaricide susceptibility and detoxifying enzyme activities in field-collected resistant and susceptible strains of *Tetranychus urticae*. Pest Manag. Sci..

[22] R Core Team (2026). R: A Language and Environment for Statistical Computing.

[23] Nawani N., Kapadnis B., Das A., Rao A., Mahajan S. (2002). Purification and characterization of a thermophilic and acidophilic chitinase from *Microbispora* sp. V2. J. Appl. Microbiol..

[24] Chang S.C., Shih H.T., Lu K.H. (2019). Antifungal effect and chitinase activities of the froth of spittlebug *Poophilus costalis* (Walker) (Hemiptera: Cercopoidea: Aphrophoridae). J. Asia-Pac. Entomol..

[25] McLaughlin B., Weiss J.B. (1996). Endothelial-cell-stimulating angiogenesis factor (ESAF) activates progelatinase A (72 kDa type IV collagenase), prostromelysin 1 and procollagenase and reactivates their complexes with tissue inhibitors of metalloproteinases: A role for ESAF in non-inflammatory angiogenesis. Biochem. J..

[26] Benjakul S., Morrissey M.T. (1997). Protein hydrolysates from Pacific whiting solid wastes. J. Agric. Food Chem..

[27] Weisburg W.G., Barns S.M., Pelletier D.A., Lane D.J. (1991). 16S ribosomal DNA amplification for phylogenetic study. J. Bacteriol..

[28] Nurk S., Bankevich A., Antipov D., Gurevich A., Korobeynikov A., Lapidus A., Prjibelsky A., Pyshkin A., Sirotkin A., Sirotkin Y. (2013). Assembling genomes and mini-metagenomes from highly chimeric reads. Proceedings of the 17th Annual International Conference on Research in Computational Molecular Biology (RECOMB 2013), Beijing, China, 7–10 April 2013.

[29] Li W., O’Neill K.R., Haft D.H., DiCuccio M., Chetvernin V., Badretdin A., Coulouris G., Chitsaz F., Derbyshire M.K., Durkin A.S. (2021). RefSeq: Expanding the Prokaryotic Genome Annotation Pipeline reach with protein family model curation. Nucleic Acids Res..

[30] Jain C., Rodriguez-R L.M., Phillippy A.M., Konstantinidis K.T., Aluru S. (2018). High throughput ANI analysis of 90K prokaryotic genomes reveals clear species boundaries. Nat. Commun..

[31] Rawlings N.D., Barrett A.J., Thomas P.D., Huang X., Bateman A., Finn R.D. (2018). The MEROPS database of proteolytic enzymes, their substrates and inhibitors in 2017 and a comparison with peptidases in the PANTHER database. Release 12.4. Nucleic Acids Res..

[32] Dybas R.A. (1989). Abamectin use in crop protection. Ivermectin and Abamectin.

[33] Nauen R., Smagghe G. (2006). Mode of action of etoxazole. Pest Manag. Sci..

[34] Bisutti I., Hirt K., Stephan D. (2015). Influence of different growth conditions on the survival and the efficacy of freeze-dried *Pseudomonas fluorescens* strain Pf153. Biocontrol Sci. Technol..

[35] Özkan M., Dilek F.B., Yetis Ü., Özcengiz G. (2003). Nutritional and cultural parameters influencing antidipteran delta-endotoxin production. Microbiol. Res..

[36] El-Bendary M.A. (2006). *Bacillus thuringiensis* and *Bacillus sphaericus* biopesticides production. J. Basic. Microbiol..

[37] Ughy B., Nagyapati S., Lajko D.B., Letoha T., Prohaszka A., Deeb D., Der A., Pettko-Szandtner A., Szilak L. (2023). Reconsidering dogmas about the growth of bacterial populations. Cells.

[38] Lachhab K., Tyagi R., Valéro J. (2001). Production of *Bacillus thuringiensis* biopesticides using wastewater sludge as a raw material: Effect of inoculum and sludge solids concentration. Process Biochem..

[39] Poria V., Rana A., Kumari A., Grewal J., Pranaw K., Singh S. (2021). Current perspectives on chitinolytic enzymes and their agro-industrial applications. Biology.

[40] Stones D.H., Krachler A.M. (2016). Against the tide: The role of bacterial adhesion in host colonization. Biochem. Soc. Trans..

[41] Vu K.D., Yan S., Tyagi R.D., Valéro J.R., Surampalli R.Y. (2009). Induced production of chitinase to enhance entomotoxicity of *Bacillus thuringiensis* employing starch industry wastewater as a substrate. Bioresour. Technol..

[42] Willis J.H. (2010). Structural cuticular proteins from arthropods: Annotation, nomenclature, and sequence characteristics in the genomics era. Insect Biochem. Mol. Biol..

[43] Fineran P.C., Blower T.R., Foulds I.J., Humphreys D.P., Lilley K.S., Salmond G.P. (2009). The phage abortive infection system, ToxIN, functions as a protein–RNA toxin–antitoxin pair. Proc. Natl. Acad. Sci. USA.

[44] Parr R.J., Santin Y.G., Ratkevičiūte G., Caulton S.G., Radford P., Jenkins M., Doyle M.T., Mead L., Silale A., van den Berg B. (2025). A porin-like protein used by bacterial predators defines a wider lipid-trapping superfamily. Nat. Commun..

[45] Veliz E.A., Martínez-Hidalgo P., Hirsch A.M. (2017). Chitinase-producing bacteria and their role in biocontrol. AIMS Microbiol..

[46] Goris J., Konstantinidis K.T., Klappenbach J.A., Coenye T., Vandamme P., Tiedje J.M. (2007). DNA–DNA hybridization values and their relationship to whole-genome sequence similarities. Int. J. Syst. Evol. Microbiol..

